# Where does resiliency fit into the residency training experience: a framework for understanding the relationship between wellness, burnout, and resiliency during residency training

**Published:** 2019-03-13

**Authors:** Liora Berger, Nishardi Waidyaratne-Wijeratne

**Affiliations:** 1 Department of Psychiatry, Queen’s University, Ontario, Canada

## Abstract

**Background:**

Medical literature reports high rates of burnout among medical professionals. The terms wellness and resilience are often used interchangeably in the solution focused discussions. By confusing the terminology, the distinct role of resilience within residency training may be ill-appreciated.The objective of this paper is to define wellness, burnout, and resiliency and detail a proposed framework to explain how these terms manifest within residency training.

**Methods:**

MEDLINE and EMBASE searches were performed using the key words “resilience,” “residency,” “wellness,” and “burnout.” The search was limited to English language articles published between 2003-2017.

**Results:**

The authors propose a framework based on the literature review. This work supports the implementation of resilience-based interventions that enable residents to engage with workplace adversity in a healthy way, acquire skills during the process and avoid resource depletion and, ultimately, burnout.

**Conclusion:**

This framework can be used by residency programs to promote educators and residents’ understanding of the unique role of resilience within residency, and the importance of differentiating between wellness and resilience initiatives. Future research is required to study the utility of this framework.

Deepthi is a first-year general surgery resident.

She was always a diligent student who was commended for her leadership qualities. Deepthi completed an undergraduate degree in biomedical sciences, a master’s degree in public heath, and four years of medical school. She developed a passion for surgery during clerkship where she was encouraged by supportive attending physicians.

Deepthi was required to relocate for her general surgery residency training, with her loyal dog Rusty as the only familiar face in town. Over the first few weeks of residency, she spent many nights on call and struggled to regulate her sleep schedule. Additionally, it was a constant challenge to find someone who could take her dog out for a walk when she was required to be in the hospital for long shifts. With time, she began to feel comfortable with her co-residents, nursing staff, and attending surgeons. Just when Deepthi developed a routine, it was time to switch to another rotation with different hours, work requirements, expectations, and staff preferences.

Mid-way through her first year, Deepthi was on an emergency medicine rotation. On this busy shift, she was seeing a patient with ascites who required paracentesis. She had performed this skill during medical school and felt comfortable attempting the procedure under supervision. After the procedure, the supervisor critiqued her “sloppy technique,” in light of surgical residents supposedly being “procedure gurus!” Deepthi then continued her shift, but could not help but dwell on each aspect of her procedure that needed improvement. She felt embarrassed, incompetent, and a sense of shame due to her unsatisfactory skills.

## Introduction

Medical culture has long emphasized that doctors should be selfless, and defer their own personal needs for the good of their patients.^1^ It is therefore expected that the path to becoming an independent practitioner will be difficult. However, the Canadian residency training experience has been evolving in a slightly different direction. The 2015 CanMEDS Physician Competency Framework delineates that in order to be a professional, one must have a commitment to self. This includes resilience, a reflective approach to practice, and a responsibility to self-care.^2^ Residency is the training period where the specific skills and dispositions towards work-life balance and the self-care of a working professional are established.^3^^-5^ This is a good time to intentionally teach healthy patterns around self-care that may be unique to the new role in the new environment. Based on updated national accreditation standards, residency training programs are encouraged to implement novel curricula to improve a resident’s capacity for self-regulation. The Resident Doctors of Canada have developed a resiliency curriculum that promotes self-regulation. Based on the National Defense’s Road to Mental Readiness, they teach residents to identify early signs of distress, and promote a toolkit of practical skills to better cope with adversity.^6^ While their focus is on resilience, it is common within medical literature for the terms wellness and resilience to be used interchangeably. By confusing the terminology, the distinct role of resilience within the residency training experience may be ill appreciated.

In this article we will define concepts of wellness, burnout, and resiliency based on a review of the medical literature. MEDLINE and EMBASE searches were performed using the key words “resilience,” “residency,” “wellness,” and “burnout.” The search was limited to English language articles published between 2003-2017. Most of the literature reviewed was specific to medical residents, but some studies on medical students and practicing physicians have been included where appropriate. We then propose a framework intended to aid in the understanding of how these unique, but interconnected elements of wellness, burnout, and resiliency can be understood within the residency training experience. The specific focus will be on resiliency and the importance of fostering these skills.

## How is resident wellness defined?

Wellness is more than a lack of impairment.^3^ It is a dynamic process involving self-awareness^3,^^7^ that results in healthy choices. It encompasses a balance between the physical, emotional, intellectual, social, and spiritual realms.^3^ Wellness provides a sense of accomplishment, satisfaction, and belonging, and offers protection from the unique demands of medical training and beyond.^3^ Measuring wellness can be difficult because there is no succinct global tool that accounts for all aspects of wellness.^8^ Stress, fatigue, time management, nutrition, activity, relationships, and the workplace environment can all affect physician wellness.^9^

## What is burnout?

Burnout is a maladaptive syndrome specific to the workplace that results from chronic work stress.^10^^-12^ According to Maslach et al., burnout has three dimensions: emotional exhaustion, a feeling of one’s energy being depleted due to excessive psychological and emotional demands; depersonalization, a tendency to be detached from others, and view others in an impersonal or cynical manner; and a diminished sense of personal accomplishment, or feelings of inadequacy in the work environment.^13^ The current “gold standard” for burnout assessment is the Maslach Burnout Inventory.^13^^,^^14^ When using this clinically validated tool, a score in the high range for emotional exhaustion or depersonalization is considered indicative of clinically significant burnout.^15^

Burnout has been associated with a lack of control, loss of self-efficacy, and powerlessness within the hierarchical culture of medicine.^8^ This syndrome leads to frustration at work, detachment from the profession and a loss of compassion.^12^ Additionally, resident burnout is associated with depression and problematic patient care.^16^

## What does it mean to be resilient?

Resilience has been defined in many ways, but, in essence, it is to thrive in the face of adversity, rather than to merely survive. It involves engaging with the harsh realities of the workplace in a healthy way such that goals are achieved at minimal physical and psychological cost.^17^ A resilient individual “bounces back” and acquires skills during the process, such as problem solving and reflective learning.^7^^,18^ Resilience was initially thought of as an inherited, stable personality trait,^1^^,18^ but research suggests that it is dynamic, relational, and temporal.^18^ This supports the implementation of resilience-based interventions which promote acquiring specific skills.^17^^,^^19^^-^^21^

Epstein notes that before an individual begins enacting resiliency strategies, he or she must have a sense of self-awareness and be able to self-monitor.^17^ This involves gaining insight into one’s ability to accept limits, acknowledge uncertainty, recognize errors, and problem solve.^1^ Self-care, a component of wellness, is important to maintain resilience. Self-care includes setting boundaries and recognizing the need to take time for oneself in the form of outside-of-work activities. While it does not address the source of stress, it promotes a sense of work-life balance, which sets a good foundation for learning and implementing resiliency skills. However, focusing only on wellness can become a survival strategy.^17^ By developing and maintaining resiliency skills, residents will cultivate healthy habits that enable them to embrace future challenges and manage difficult situations based on previous experiences.^17^ Physicians who exhibit resilience have a better sense of overall wellbeing, provide better quality of care for their patients, and contribute to an overall decrease in health care costs.^22^

## A framework for understanding wellness, burnout, and resiliency within the residency training experience

### A unique set of baseline attributes

Each physician enters the residency training experience with their own unique set of baseline attributes and support systems. These serve as protective factors and are necessary for the process of developing resilience to occur.^18^^,^^23^^,24^ Commonly cited factors include a sense of personal worthiness, belief in one’s own self efficacy, trust, hope, critical thinking, problem solving, positive social orientation, and a sense of humor.^19^ While the presence of each of these factors is important, they are discrete, contextual, and lead to varying outcomes depending on the circumstance.^25^

### The residency training experience

The residency training experience is different from medical school, and different from independent practice. Residents are transitioning from learner to health care provider, and from learner to teacher while remaining on the lower end of the physician hierarchy. Residents may struggle with uncertainty of expectations, transferring knowledge into practice, increased on-call responsibilities, and experiencing patient death.^26^^,^^27^ Situational stressors such as acute or chronic sleep deprivation, acute resuscitation decisions, and quality of supervision are all elements of the residency experience.^26^ Additionally, residents may be asked to participate in care that they feel is suboptimal or otherwise unethical, yet under pressure from perceived and likely unspoken expectations, feel obligated to follow, what seem to them to be, orders. They have an expected responsibility to patient care, with a perceived limited ability to influence care decisions.^10^

The structure of residency training includes the expectation of service excellence and curative competence,^20^ in the context of a rotational model of clinical training. This constant team re-arrangement may impede the development of meaningful working relationships and limit a resident’s support network.^28^ Residents also have a lack of control over their schedules and working conditions, which is a strong predictor of burnout in practicing physicians.^29^ Personal stressors such as familial obligations, isolation due the time demands of one’s workload, and medical school debt are additional sources of stress among resident physicains.^30^

The Job Demands-Resources (JD-R) model suggests that job resources interact with job demands to affect employee health and wellbeing.^31^ In line with this model, when job demands require high effort that is not possible in the context of the available job resources, these demands become stressors and will likely lead to burnout.^31^^,32^ During times of stress, a resident may want to seek help, but experience barriers to disclosure including career implications, stigma, and professional standing.^33^ A resident may instead focus on the end goal and view some of the demands of residency as necessary elements of this rite of passage. Unfortunately, as training progresses, residents may be faced with the risk of job uncertainty after graduation. Overwhelming work requirements coupled with a lack of job resources can further burden the experience of training to become an independent practitioner.

### Facing adversity

During residency, as in many other professions there will be times when the resident must face adversity. Adversity refers to a change in routine, a disruption to one’s usual encounters, or a challenge that must be overcome.^19^ For residents, this includes adapting to a new set of roles and responsibilities while trying to learn and master new techniques. In the medical setting additional adverse events such as a medical error, complaint, litigation, intimidation, and harassment may occur.^9^ These negative experiences tend to threaten a resident’s physical, emotional, or financial well-being, which can elicit the body’s alarm reaction resulting in the physical manifestations of stress.^18^

**Figure 1:**
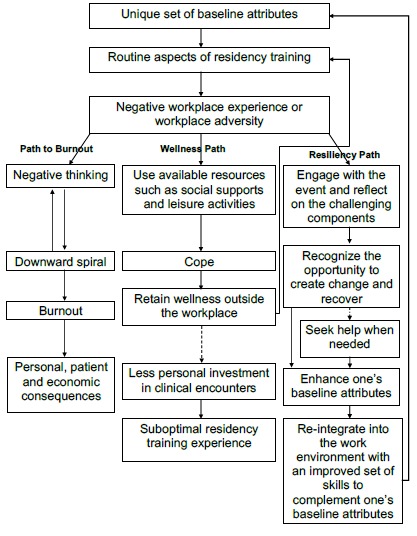
A framework for understanding the relationship between wellness, burnout, and resiliency, during residency training

When facing adversity, each resident has a unique way of responding to the negative experience. As depicted in Figure 1, the possible responses can be divided into three broad paths: the path to burnout, the wellness path, and the resiliency path. While these paths are interconnected, they can be viewed as distinct. This outlook enables residents to visualize the potential consequences of their own negative experience.

### The path to burnout

In the context of a negative experience, a resident may engage in negative thinking, which, if occurring repeatedly, can lead to a downward spiral eventually resulting in burnout. In these situations, residents may attribute the adverse event purely to an internal cause, thus experiencing a sense of shame.^34^ According to the Conservation of Resources Theory, ongoing low level resource depletion from either actual or perceived loss of resources leads to burnout.^35^ In the residency training experience, these resources may include high work demands, low organizational supports, problematic supervisory relationships, few learning opportunities, and others.^36^ These can lead to increasing doubts regarding self-efficacy, less engagement in daily work and draining of personal resource reservoirs.^36^ By depleting these resources, residents will likely experience clinical burnout.^15^ In 2008-2009, a large cross-sectional study done in the United States revealed that 51.5% of the 16,394 surveyed Internal Medicine residents met criteria for burnout.^37^ A few years earlier, Martini reported similar findings, with no significant difference between specialties.^11^ Preliminary reports from the 2017 Canadian Medical Association National Physician Health survey, revealed that 38% of residents across Canada reported high burnout rates.^38^

Burnout has devastating consequences on the individual, patient, and community.^13^ Prins et al. reviewed the literature on burnout in medical residents and found associations between resident burnout and health problems such as depression, somatization, alcohol abuse, and general anxiety.^39^ With regards to patient safety, Shanafelt et al. found that among the 7905 surgeons surveyed, 8.9% reported concern of a major medical error in the past three months.^40^ Burnout can also have an impact on the larger community, as residents experiencing burnout may be absent more often, or leave the workforce altogether. In North America, based on the time and money spent to train the practitioner, this can amount to an economic loss of $497,000.^22^^,^^41^

### The wellness path

After a negative experience in the work environment, a resident has the opportunity to remove themselves from the situation. They may use their available resources including social supports, leisure activities or meditation to take their mind off the demands and challenges of their work environment. This enables residents to cope with hardship. Coping in this manner demonstrates a sense of hardiness, a personality trait that allows an individual to endure adversity.^19^ Coping preserves wellness, which in the short term enables a resident to return to work and perform daily required activities. Within the pathway, the adverse event has not enabled the resident to develop any skills that can be used if a similar negative experience were to recur. Consistent with the JD-R model, the Conservation of Resources theory would suggest, that if resource depletion continued to occur without intervention, active coping and work engagement would decline.^31^^,^^35^^,36^ In this situation, a resident would likely enjoy self-care activities more than daily responsibilities that come with the expectation of insurmountable adversity. This may result in withdrawal from professional activities and less time spent engaged in learning and patient care. Ultimately, this has the potential to be detrimental to the quality of a resident’s training.

### The resiliency path

In the face of adversity, a resident has the ability to engage with the harsh reality of the workplace and reflect on the challenging components rather than removing themselves from the situation. Instead of being occupied with negative thoughts, or disregarding the event, the resident can recognize the event as an opportunity for growth. Resiliency involves recovering from these events with minimal physical or psychological harm and creating change so that the adverse event will not cause the same pattern of stress in the future.^18^ This will prevent withdrawal from practice, and enable the resident to re-integrate into the work environment and function at an optimal level.

When reserves are depleted, it may be difficult to utilize resilience tools that have been effective previously. An aspect of resiliency is self-reflecting and recognizing when to ask for help.^6^ According to Canadian national accreditation standards for residency training, all residents must be aware of and have access to confidential services to manage stress.^42^ While informal help from peers is appropriate at earlier stages of distress, it is important to seek formal help should the situation deteriorate.^6^ If residents are aware that asking for help is an element of resilience, rather than a display of weakness, they may be more inclined to utilize available resources.

Deepthi realised that she had a few options of how to respond to her negative experience: she could continue to think negatively of herself, her career choice and her future; forget about this experience, go home, walk the dog and start fresh tomorrow; or consider how this negative experience can best serve as a learning opportunity.

Deepthi recognized that while she had practiced the paracentesis procedure in medical school, there were a few factors that affected her performance. Firstly, it had been a long time since performing this skill and she did not have a clear plan in mind. Secondly, the tools used in this hospital were slightly different, which affected the flow of the procedure and her confidence. Lastly, she felt rushed by the supervisor who had other responsibilities to attend to. Deepthi spent that evening reviewing indications for paracentesis, procedural steps involved, and watched online videos to solidify the concepts. Anticipating another situation that may be similar in the future, she planned for how she could handle this. She came up with simple ideas such as familiarizing herself with the equipment, and mentioning her level of comfort to the supervisor prior to entering the patient’s room. She felt stronger from this experience and looked forward to her next opportunity to use her refined skills.

A few weeks later while on call, Deepthi was required to insert a nasogastric tube, which she had not done for a while. Remembering her previous experience, she located all of the necessary equipment and asked the senior resident if he was available to provide supervision. She went over the steps with the senior and received some helpful tips prior to entering the patient’s room. Deepthi was able to use her previous negative experience to improve the quality of this experience. She bounced back from adversity, and the outcome of her response exemplifies the value of resilience during residency training.

### Conclusion

With the evolution of accreditation standards, and an increased emphasis on professional competency, there is an expectation that residency programs place a greater focus on the health and well-being of their learners.^2^ Many programs focus on preventing burnout through stress reduction and work-life balance, rather than exploring hardship as an opportunity for learning. Some residency training programs have implemented initiatives to enhance resident resilience,^43^^-^^45^ but at times the terminology can be confusing as the terms resilience and wellness are often used interchangeably

This proposed framework (Figure 1) can be used by residency programs to promote educators and residents’ understanding of the unique role of resilience within residency, and the importance of differentiating between wellness and resilience initiatives.

Future research into adverse events experienced in each specialty may enable interventions that are specialty-relevant. Additionally, residents spend a great deal of time with medical students and thus residents’ resilience, will be directly modeled to those in early stages of medical training.

With the high prevalence of depression, suicide, and burnout among resident physicians, it is important for residents to develop the ability to engage with the demands of residency training in a manner that promotes professional growth. This work supports the implementation of resilience-based interventions that have the ability to be protective by enabling a resident to engage with workplace adversity in a healthy way rather than depleting their resources and experiencing burnout. Further research is necessary to determine if this framework provides residents and educators with an understanding of the value of resilience within the residency training experience.
